# Supplementary material for: Tead1a Initiates Transcriptional Priming Through the TEAD1a/YAP‐Notch1‐Spi1/Cebpα Axis to Promote Neutrophil Fate
Source: Adv Sci (Weinh). 2025 Aug 28;12(41):e05441. doi: 10.1002/advs.202505441 (PMC12591127; doi:10.1002/advs.202505441)
Supplement: Supplementary file 4 — Supplementary Dataset [file ADVS-12-e05441-s004.zip › 3-6.zip!/3-6/02-6.cluster/cluster17/Enrich/ego_all.pdf]

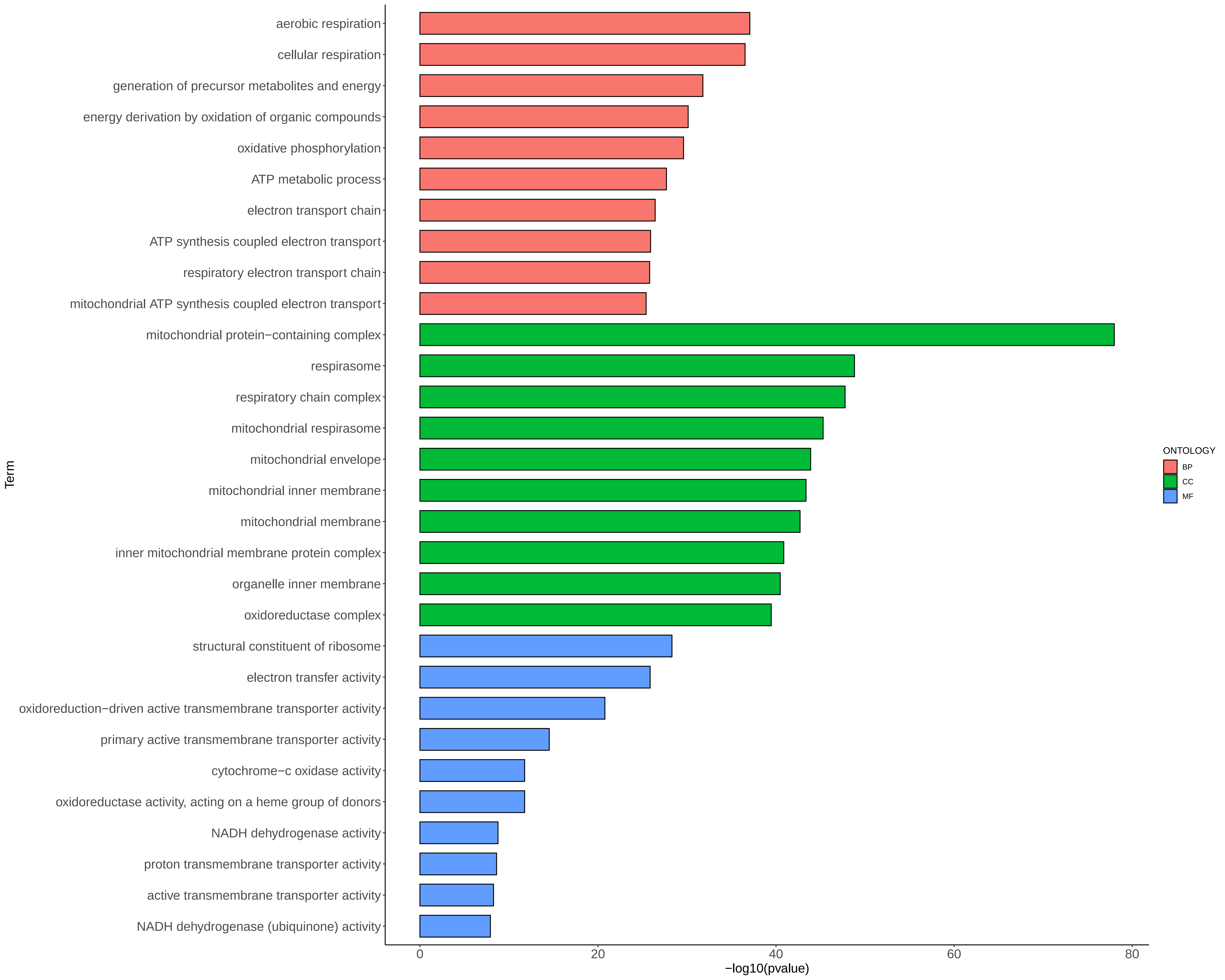

aerobic respiration
cellular respiration
generation of precursor metabolites and energy
energy derivation by oxidation of organic compounds
oxidative phosphorylation
ATP metabolic process
electron transport chain
ATP synthesis coupled electron transport
respiratory electron transport chain
mitochondrial ATP synthesis coupled electron transport
mitochondrial protein−containing complex
respirasome
respiratory chain complex
mitochondrial respirasome
mitochondrial envelope
mitochondrial inner membrane
mitochondrial membrane
inner mitochondrial membrane protein complex
organelle inner membrane
oxidoreductase complex
structural constituent of ribosome
electron transfer activity
oxidoreduction−driven active transmembrane transporter activity
primary active transmembrane transporter activity
cytochrome−c oxidase activity
oxidoreductase activity, acting on a heme group of donors
NADH dehydrogenase activity
proton transmembrane transporter activity
active transmembrane transporter activity
NADH dehydrogenase (ubiquinone) activity
Term
0
20
40
60
80
−log10(pvalue)
ONTOLOGY
BP
CC
MF
